# Reducing Depression Through an Online Intervention: Benefits From a User Perspective

**DOI:** 10.2196/mental.4356

**Published:** 2016-01-08

**Authors:** Dimity A Crisp, Kathleen M Griffiths

**Affiliations:** ^1^Centre for Applied PsychologyFaculty of HealthUniversity of CanberraCanberraAustralia; ^2^National Institute for Mental Health ResearchThe Australian National UniversityCanberraAustralia

**Keywords:** Internet interventions, depression

## Abstract

**Background:**

Internet interventions are increasingly being recognized as effective in the treatment and prevention of mental health conditions; however, the usefulness of such programs from the perspective of the participants is often not reported.

**Objective:**

This study explores the experiences of participants of a 12-week randomized controlled trial of an automated self-help training program (e-couch), with and without an Internet support group, targeting depression.

**Methods:**

The study comprised a community sample of 298 participants who completed an online survey both prior to and on completion of an intervention for preventing or reducing depressive symptoms.

**Results:**

Overall, participants reported a high level of confidence in the ability of an online intervention to improve a person’s understanding of depression. However, confidence that a website could help people learn skills for preventing depression was lower. Benefits reported by participants engaged in the intervention included increased knowledge regarding depression and its treatment, reduced depressive symptoms, increased work productivity, and improved ability to cope with everyday stress. A minority of participants reported concerns or problems resulting from participation in the interventions.

**Conclusions:**

The findings provide consumer support for the effectiveness of this online intervention.

**Trial Registration:**

International Standard Randomized Controlled Trial Number (ISRCTN): 65657330;http://www.isrctn.com/ISRCTN65657330 (Archived by WebCite at http://www.webcitation.org/6cwH8xwF0)

## Introduction

Growing numbers of people are turning to the Internet as a preferred method for obtaining health-related information, or as an option for accessing therapeutic interventions [1]. In the area of mental health, Internet-based interventions for depression have undergone significant development [2]. Importantly, as use and service options have expanded, research has demonstrated the efficacy of online programs in reducing depressive symptoms [3-10], improving depression literacy [11,12], and decreasing stigma [11-13]. However, while increased interest in online participation appears indicative of the acceptance of Internet-based interventions, the benefits of these programs from the perspective of the participants is often not reported.

Consumer satisfaction is integral in assessing the quality of any health service delivery. While the effectiveness of interventions in reducing symptoms is critical, the experience and satisfaction of participants has important implications for treatment outcomes, continuation of treatment (adherence), and re-connection with a service at a latter point of need [14,15]. Consumer satisfaction reports are increasingly being used to assess the quality of mental health services [14,16-19]. Some research has also investigated the relationship between consumer satisfaction and objective mental health outcome measures [20]. However, to date, consumer feedback has primarily been utilized in the development and evaluation of community-based mental health services [21].

Some researchers have investigated consumer satisfaction with e-mental health programs. Proudfoot and colleagues [22] investigated community attitudes and the acceptability of mobile phones for self-monitoring of symptom severity and obtaining self-help for depression, anxiety, and stress, and obtained positive feedback. High levels of general satisfaction with Internet-based treatment has also been reported in the context of randomized controlled trials (RCTs) conducted for various mental health conditions [23-25]. One study [25] investigating an Internet based intervention for depression found that, on average, participants reported feeling very confident that the “treatment would be successful at teaching them techniques for managing their symptoms”; and a high level of confidence in “recommending the treatment to a friend with depression” (p21). However, largely this is an area within e-mental health services research that has not undergone detailed investigation.

Evaluations of the WellBeing trial [26,27] found that exposure to a 12-week automated training program comprising psycho-education and cognitive behavioral and interpersonal therapy (e-couch) used both alone and in combination with an Internet support group (ISG) produced significant reductions in depressive symptoms at 6- and 12-month follow-ups [27]. In addition, the automated training program was associated with immediate improvements in self-esteem and empowerment relative to control participants, and when combined with the ISG, 6-month improvements in perceived quality of life [28]. However, the subjective experience of the intervention participants in the WellBeing trial was not reported.

The aims of the present study are to (1) explore the level of consumer confidence in online e-mental health programs both prior to and following participation in the trial; (2) investigate the benefits and changes in behavior reported by participants following participation in the trial; and (3) identify any problems encountered by participants. Further, given that publicly available online mental health (depression) interventions are most likely to be accessed by individuals self-identifying as being depressed, comparisons were made between those reporting that they currently suffer from depression (at baseline) with those that did not.

## Methods

This study was approved by The Australian National University Human Research Ethics Committee (Protocol 2007/2259) and the WellBeing trial described in this paper was registered with the Controlled Clinical Trials registry (ISRCTN65657330). The current paper contains only a brief description of the methodology specific to the present study as the complete WellBeing trial protocol has been published previously [26,27].

### Participants

The study comprised 298 adults aged 18 to 65 years recruited to the WellBeing trial between August 2008 and May 2009. Participants were recruited through a screening survey posted to 70,000 adults randomly selected from the electoral rolls of 8 Australian electoral divisions (4 rural and 4 metropolitan). Participants were informed that the trial was designed to investigate “the usefulness of self-help Internet programs for improving emotional well-being and preventing or reducing the symptoms of depression” [26]. To be eligible for the trial participants required a Kessler Psychological Distress (K10) score greater than 22, and access to the Internet. Respondents were excluded if they were currently (1) receiving treatment from a mental health professional or participating in a mutual support group; (2) participating in another research project at the lead investigator’s (KG) research center; and (3) reported current or past experience with or diagnosis of psychosis, schizophrenia, or bipolar disorder.

### Procedure

The study employed a longitudinal RCT design. Data pertaining to participant confidence in online interventions and satisfaction were collected via self-report questionnaires administered at baseline (one week prior to commencement of the intervention), and at the conclusion of the 12-week intervention period.

### Interventions

Participants were randomly allocated to receive one of four conditions: an ISG, which utilized a moderated bulletin board format to facilitate discussions between participants on topics primarily related to depression; a depression Internet training program (e-couch) comprising a depression literacy component and online versions of cognitive behavior therapy, interpersonal therapy, applied relaxation, and physical activity programs; a combination of the two (e-couch and ISG); or an attention control website (HealthWatch). HealthWatch comprised 12 online modules, each containing the following components: (1) a series of questions participants were asked to consider on topics potentially related to depression and well-being (eg, nutrition), and (2) online health information about a topic that may be related to well-being but did not address depression specifically (eg, environmental health). The content of each intervention has been described in further detail elsewhere [26,27].

Randomization was conducted by the trial statistician using a stratified block design procedure, with a fixed block size of 4. Stratification variables were level of psychological distress, gender, age, and location of residence [27]. The number of participants completing the baseline questionnaire and commencing the intervention following randomization was as follows: ISG (n=77), e-couch (n=74), e-couch and ISG (n=73), and HealthWatch (n=74).

### Measures

This study explores participant confidence in the effectiveness of online interventions (collected prior to and after the intervention), and problems encountered, self-reported benefits, and behavior change following the intervention.

#### Intervention Benefits

Immediately following the intervention trial period, participants were asked to respond to 14 items reporting the extent to which the website helped them in areas such as “being productive at work” and “reducing the emotional pain they were experiencing”; and the extent to which the website helped them to “discuss subjects that I felt unable to discuss before”, “feel encouraged and supported emotionally”, “feel less isolated and lonely”, “feel proud of myself for helping others”, “learn more about depression and its treatment”, or “seek professional help for my depression”. Items were developed for the purpose of the study based on items developed in a UK study of depression ISG users [29], and the Consumer Reports Effectiveness Scale (CRES-4) [17,30]. Participants responded to each statement on a 5-point scale from 1(*strongly agree/made things a lot better*) to 5(*strongly disagree/made things a lot worse*). The scale was then dichotomized as 0 (*neither/no difference, disagree/made things somewhat worse, strongly disagree/made things a lot worse*), 1 (*agree/made things somewhat better, strongly agree/ made things a lot better*) to assess the proportion of participants reporting benefit in each area.

#### User Confidence

At each assessment point participants rated their confidence that a website could both “help people understand depression better” and “help people learn skills for preventing depression”. Participants responded 0 (No) or 1 (Yes) to each statement.

#### Behavior Change

Participant self-reported behavior change was assessed across the sample using 4 items. Participants responded 0 (No) or 1(Yes) to indicate if they had “given advice about depression to someone else”, “sought help from a health professional”, “sought more information”, or “tried a self-help treatment”.

#### Problems Encountered

Following the intervention trial period, participants were asked to indicate if they had encountered any problems as a result of using the website or participating in the trial. Responses were recorded as 0 (No) or 1 (Yes). Items were developed for the purpose of the study. The survey was tailored to the types of problems that may have been encountered in each trial arm. Items in the survey of participants exposed to the HealthWatch (control) or e-couch (including combined condition) websites were “Feeling bored”, “Feeling frustrated”, “Feeling more anxious”, and “Finding the program too impersonal”. Items received by ISG participants (including combined condition) were “Feeling annoyed or upset by the comments made by other members on the board”, “Feeling frustrated that I could not meet other members of the board in person”, “Feeling upset that I couldn't help other board members more”, and “Feeling very anxious about other members on the board”.

### Statistical Analysis

All primary comparisons were conducted using chi-square analyses. Post-hoc comparisons identifying significant differences between the four intervention groups were based on the standardized residual for each cell. Values greater than ±1.96 indicate a difference at a *P* value of .05, and values greater than ±2.58 indicate a difference at a *P* value of .01.

## Results

The numbers of participants in each condition at each wave of data collection (baseline and post-test) are shown in Table 1. At post-test, drop-out was significantly lower in the HealthWatch control condition compared to the other conditions. Few significant differences were found between completers and non-completers on measures of depression or demographic characteristics [27].

**Table 1:** Number of participants in each condition at baseline and post-test.

	Total sample, N	Intervention condition, n (%)			
		HealthWatch	e-couch	ISG	e-couch and ISG
Baseline	298	74 (24.8)	74 (24.8)	77 (25.8)	73 (24.5)
Post	231	71 (30.7)	59 (25.5)	53 (22.9)	48 (20.8)

### Confidence in Online Interventions

Across the sample it appeared that confidence in a website as a tool to improve depression literacy was high (Table 2). At baseline (prior to interacting with the website), 83.6% (249/298) of participants were confident that a website could help people understand depression better. Confidence in the ability of a website to help people learn skills for preventing depression was less certain across the sample at baseline, with only 48.3% (144/298) of people making the endorsement.

**Table 2:** Number of people confident that a website could improve depression literacy or be used as a prevention tool.

		Depression literacy^a^		Prevention tool^b^	
		Baseline	Post	Baseline	Post
Total sample, n/N^c^ (%)		249/271 (91.9)	195/212 (92.0)	144/235 (61.3)	135/203 (66.5)
**Intervention condition, **n/N (%)					
	HealthWatch	62/71 (87.3)	62/67 (92.5)	38/62 (61.3)	40/64 (62.5)
	e-couch	61/67 (91.0)	51/56 (91.1)	40/63 (63.5)	37/54 (68.5)
	ISG	68/70 (97.1)	42/48 (87.5)	37/61 (60.7)	27/46 (58.7)
	ISG and e-couch	58/63 (92.1)	40/41 (97.6)	29/49 (59.2)	31/39 (79.5)
	*P* ^d^	.200	.972	.372	.190
**Current depression,** **n/N (%)**					
	Yes	164/184 (89.1)	131/141 (92.9)	91/160 (56.9)	87/134 (64.9)
	No	82/84 (97.6)	62/69 (89.9)	52/72 (72.2)	47/68 (69.1)
	*P* ^d^	.019	.446	.026	.551

^*a*^Confident that a website could help people understand depression better.

^*b*^Confident that a website could help people learn skills for preventing depression.

^*c*^N values vary due to missing data.

^d^Chi-square significance level for the test of difference between groups.

Following the intervention period this indicator of confidence remained relatively stable with no significant change in the proportion of respondents making these endorsements. No significant differences were found based on intervention group. There were significant differences in the responses of participants who did and did not report depression at baseline. At baseline, participants reporting current depression were significantly less likely than those not reporting depression to be confident that a website could either improve people’s understanding of depression (*P*=.019) or teach people skills for preventing it (*P*=.026). However, this significant difference was not maintained following the intervention. Following the intervention participants were asked to report on the usefulness of the website. Of the 226 respondents to the question, 76.5% (173/226) of people indicated they found the website useful or very useful. There were no significant differences between any of the conditions, or on the basis of self-reported depression at baseline (Table 2).

### User-Reported Benefits

The most frequently endorsed benefit of participating in the trial by participants across the sample was one of increasing depression literacy (Table 3). Across the total sample, 76.2% (170/223) of respondents indicated that the website helped them to “learn more about depression and its treatment”. In particular, this benefit was strongly endorsed by participants in the e-couch and combined e-couch and ISG condition; participants in the ISG condition were significantly less likely to report that the website helped them to learn more about depression and its treatment when compared to the e-couch and combined ISG in combination with e-couch conditions (*P*<.05). No significant differences were found between those participants self-reporting current depression at baseline and those who did not.

**Table 3:** Reported benefits of engaging in the online interventions, by intervention condition.

		Total sample, n/N^a^ (%)^b^	Intervention condition				*P* ^c^
			HealthWatch, n/N (%)^b^	e-couch, n/N (%)^b^	ISG, n/N (%)^b^	e-couch and ISG, n/N (%)^b^	
**The website helped me to…**							
	Discuss subjects that I felt unable to discuss before	94/220 (42.7)	22/65 (33.8)	25/58 (43.1)	26/50 (52.0)	21/47 (44.7)	.269
	Feel encouraged and supported emotionally	104/218 (47.7)	23/64 (35.9)	29/57 (50.9)	28/51 (54.9)	24/46 (52.2)	.157
	Feel less isolated and lonely	93/219 (42.5)	18/65 (27.7)	26/57 (45.6)	25/51 (49.0)	24/46 (52.2)	.033
	Feel proud of myself for helping others	74/209 (37.1)	23/62 (37.1)	14/53 (26.4)	21/49 (42.9)	16/45 (35.6)	.370
	Learn more about depression and its treatment	170/223 (76.2)	48/69 (69.6)	52/58 (89.7)	23/49 (46.9)	47/47 (100.0)	<.001
	Seek professional help for my depression	35/204 (17.2)	8/62 (12.9)	10/53 (18.9)	6/46 (13.0)	11/43 (25.6)	.309
**How much do you feel the website helped you in**							
	Being productive at work	62/209 (29.7)	14/63 (22.2)	22/55 (40.0)	8/48 (16.7)	18/43 (41.9)	.010
	Coping with everyday stress	115/218 (52.8)	25/65 (38.5)	38/58 (65.5)	22/50 (44.0)	30/45 (66.7)	.003
	Enjoying life more	96/215 (44.7)	23/65 (35.4)	31/57 (54.4)	17/50 (34.0)	35/43 (58.1)	.019
	Personal growth and understanding	125/216 (57.9)	32/64 (50.0)	38/57 (66.7)	28/50 (56.0)	27/45 (60.0)	.309
	Reducing the emotional pain you were experiencing	92/213 (43.2)	17/64 (26.6)	30/56 (53.6)	21/49 (42.9)	24/44 (54.5)	.007
	Reducing the symptoms of your depression	97/214 (45.3)	18/62 (29.0)	34/57 (59.6)	17/52 (32.7)	28/43 (65.1)	<.001
	Your ability to relate to others	105/218 (48.2)	22/65 (33.8)	35/58 (60.3)	24/50 (48.0)	24/45 (53.3)	.026
	Your self-esteem and confidence	88/217 (40.6)	20/64 (31.3)	29/57 (50.9)	20/51 (39.2)	19/45 (42.2)	.179

^*a*^N values vary due to missing data.

^b^Percentage of respondents endorsing the statement as “agree-strongly agree” or “made things a lot or somewhat better”.

^c^Chi-square test of significance for the difference between groups.

When asked how much they felt that the website had helped them in areas such as coping with stress and reducing symptoms of depression, the benefit endorsed most by participants was that of promoting personal growth and understanding, reported by 57.8% (125/216) of respondents (Table 3). Comparing benefits obtained across the different intervention groups it was found that the e-couch and combined e-couch and ISG condition participants were again significantly more likely to report that the website helped reduce symptoms of depression and increase productivity at work, the ability to cope with everyday stress, and enjoy life more, compared to the control and ISG alone conditions (significant at *P*<.05). Compared to the control condition, participants in the e-couch and combined condition were more likely to report that the website helped their ability to relate to others, and reduced their emotional pain (*P*<.05). The e-couch participants were also more likely to report that the website helped with self-esteem and confidence compared to the control group (significant at *P*<.05). Again, no significant differences were found between those participants self-reporting current depression at baseline and those who did not. See Multimedia Appendix 1 for each comparison.

When asked if they had done something different (eg, sought more information or treatment) as a result of the website, approximately 47.8% (109/228) of respondents indicated they had (Table 4). This was significantly higher amongst participants in the e-couch and combined e-couch and ISG conditions. Respondents in both conditions were more likely to report have done something different because of the website compared to the control and ISG conditions (*P*<.01). Specifically, when asked what they had done, participants in both the e-couch and the combined condition were more likely to have tried a self-help treatment compared to control and ISG conditions (significant at *P*<.01), as was the combined condition (significant at *P*<.05).

**Table 4:** Reported help-seeking actions following participation, by intervention condition, in response to the question “Have you done something different because of the website?”

	Total sample, n/N^a^ (%)^b^	Intervention conditions				*P* ^c^
		HealthWatch, n/N (%)	e-couch, n/N (%)	ISG, n/N (%)	e-couch and ISG, n/N (%)	
Yes (total)	109/228 (47.8)	17/70 (24.3)	43/59 (72.9)	13/51(25.5)	36/48 (75.0)	<.001
Yes, given advice about depression to someone else	28/228 (9.4)	4/71 (5.6)	10/58 (17.2)	5/52 (9.6)	9/47 (19.1)	.084
Yes, sought help from a health professional	16/228 (5.4)	2/71 (2.8)	5/58 (8.6)	2/52 (3.8)	7/47 (14.9)	.060
Yes, sought more information	33/228 (11.1)	6/71 (8.5)	7/58 (12.1)	8/52 (15.4)	12/47 (25.5)	.071
Yes, tried a self-help treatment	66/228 (22.1)	6/71 (8.5)	31/58 (53.4)	6/52 (11.5)	23/47 (48.9)	<.001

^a^N values vary due to missing data.

^b^Percentage of respondents endorsing the statement.

^c^Chi-square test of significance for the difference between groups.

Overall, those reporting depression at baseline were not more likely than their counterparts not reporting depression to have done something different because of participation in the program or website. However, when asked about what they had done, participants self-reporting current depression at baseline who had taken action were significantly more likely to have sought help from a professional (*P*=.022), and were more likely to have sought more information (*P*=.055). For tables representing each comparison, see Multimedia Appendices 1-3.

### User Problems

The investigation of concerns or problems encountered by participants indicated few stressors resulted from participation in the interventions (Table 5). When asked about problems resulting from the use of the e-couch or control program, 27.6% (45/162) of respondents overall reported finding the program too impersonal. While no significant difference was found between conditions, the highest endorsement figure was for the HealthWatch condition which used impersonal content, followed by e-couch and the conditions involving an ISG. Problems of “Feeling frustrated and bored” were endorsed by less than 20% of the sample; 11% (18/165) of the participants reported feeling anxious as a result of using a program. Examining differences between conditions, respondents in the combined condition were more likely to report feeling more anxious or feeling frustrated compared to the control condition (significant at *P*<.05).

**Table 5:** Problems reported by trial participants by intervention condition.

		Total sample, n/N^a^ (%)^b^	Intervention condition				*P* ^c^
			HealthWatch, n/N (%)^b^	e-couch, n/N (%)^b^	ISG, n/N (%)^b^	e-couch + ISG, n/N (%)^b^	
**Problems reported with the e-couch or HealthWatch program?**							
	Feeling bored	29/165 (17.6)	9/67 (13.4)	12/56 (21.4)	N/A	8/42 (19)	.489
	Feeling frustrated	32/166 (19.3)	7/68 (10.3)	11/56 (19.6)	N/A	14/42 (33.3)	.012
	Feeling more anxious	18/165 (10.8)	3/66 (4.5)	5/56 (8.9)	N/A	10/43 (23.3)	.008
	Finding the program too impersonal	45/162 (27.6)	23/64 (35.9)	15/55 (27.3)	N/A	7/43 (16.3)	.084
**Problems reported with the WellBeing Board?**							
	Feeling annoyed or upset by the comments made by other members on the board	8/94 (8.4)	N/A	N/A	5/51 (9.8)	3/43 (7.0)	.625
	Feeling frustrated that I could not meet other members of the board in person	16/92 (17.0)	N/A	N/A	11/49 (22.4)	5/43 (11.6)	.172
	Feeling upset that I couldn't help other board members more	27/91 (29.0)	N/A	N/A	14/49 (28.6)	13/42 (31.0)	.804
	Feeling very anxious about other members on the board	14/93 (14.7)	N/A	N/A	8/50 (16)	6/43 (14)	.783

^a^N values vary due to missing data.

^b^Percentage of respondents endorsing the statement is indicated.

^c^Chi-square test of significance for the difference between groups.

When asked about problems resulting from participation in the ISG, 29% (27/91) of respondents overall reported feeling upset that they could not help the other members of the ISG more, and 17% (16/92) reported frustration that they could not meet the other members of the ISG in person (anonymity was a rule governing participation in the ISG).

No significant differences in concerns or problems encountered were found between those participants self-reporting current depression at baseline and those who did not (see Multimedia Appendices 1-3 for tables presenting each comparison). Few participants reported being upset or annoyed by the comments of others in the ISG.

## Discussion

To our knowledge, this is the most comprehensive quantitative investigation of consumer perspectives on Internet-based depression interventions. Examining confidence and satisfaction with the online services, the benefits and changes in behavior reported by participants following participation in the trial, and problems encountered by participants, the study provides evidence to support the benefits of online psycho-education and cognitive behavior therapy programs from a consumer perspective. Overall, participants reported a high level of confidence in the ability of the online interventions to improve people’s understanding of depression, and following participation in the program a clear majority reported the interventions were useful. Participants engaged in the e-couch intervention indicated that the website helped them to learn more about depression and its treatment and helped reduce symptoms of depression, increase productivity at work, improve their ability to cope with everyday stress, and enjoy life, compared to the control and ISG conditions. A minority of participants reported concerns or problems resulting from participation in the interventions.

### Confidence in Online Interventions

The successful development and implementation of online interventions for mental health conditions, such as depression, depend on consumers’ confidence in the program and willingness to engage in this alternative treatment. As such, the present study first examined participant confidence that a website could help people understand and learn skills for preventing depression. Results found that overall confidence in the ability of a website to increase understanding of depression was high; however, only 61.3% (144/235) of participants at baseline reported confidence in a website (online intervention) as a tool to help people learn skills for preventing depression. The high level of confidence in the ability of a website to increase knowledge or literacy, particularly at baseline, is perhaps unsurprising given that the Internet is largely an information resource and the participants of this study had signed up for an online intervention trial. However, the comparatively lower confidence in websites as prevention strategies or tools is of importance since such perceptions may serve as a barrier to the use of the Internet for prevention programs for depression. Moreover, participants reporting current depression were significantly less confident in the ability of a website to either improve people’s understanding of depression or teach people skills for preventing it, compared to those not reporting depression. Despite this scepticism, the willingness of participants to participate in the study (promoted as a trial designed to improve well-being) and to try new interventions is positive. Importantly, the differences (albeit small effects) in confidence that a website could help people understand depression or learn skills for preventing depression identified at baseline between participants self-reporting depression and those that did not, were not found following the intervention. While no significant increase was found across the sample in overall confidence in the ability of an online intervention, the results reflect small increases in confidence after having completed the intervention amongst those participants reporting current depression at baseline. Expectations are likely to play a significant role in whether a consumer seeks help from an online program. Accordingly, the continued monitoring of the acceptability and perception of e-mental health initiatives and programs should be central to the continued expansion and implementation of e-mental health services. This monitoring also should include items which determine consumer perspectives on the effectiveness of online programs for treating depression as well as preventing it. It is possible that perceived treatment effectiveness would be higher than perceived preventive effectiveness among consumers. However, this requires further consideration.

### Reported Benefits of Participation

Participants reported benefits and behavior changes as a result of their participation in the trial. An increase in depression literacy was reported by over half of participants, with this benefit most strongly endorsed by those engaged with e-couch (either alone or in combination with the ISG). This is consistent with the fact that e-couch incorporates a specific depression literacy and educational component and might therefore reasonably be expected to deliver greater improvements in literacy than either an ISG or the control condition. One question of interest is whether self-reported improvements in depression literacy should be greater among the ISG participants than the control group given that depression ISGs are often used as a means of communicating information between members [31]. Here they were not. In fact, a surprising large percentage of the HealthWatch control participants perceived an improvement in their knowledge about depression and treatments after completing the condition. This may be explained by the fact that in the quiz sections of the HealthWatch condition participants were asked to think about the role of different lifestyle issues in depression (eg, humor in depression). Participants may have interpreted this as information about depression and its treatment. Further, the ISG used was an experimental group that was established for the purpose of the research trial. Research is required to investigate the perceived benefits of well-established Internet groups with large numbers of participants including those with strong depression literacy.

Participants allocated to e-couch (more than other intervention groups) reported the website as helping to reduce symptoms of depression, increase productivity at work, improve ability to cope with everyday stress and enjoy life more. Further, they reported an improved ability to relate to others and reduced emotional pain compared to control participants. These findings provide corroboration that e-couch is a helpful online intervention for depression. Specifically, these reports complement the initial evaluation of the WellBeing trial that found a significant objective reduction in depressive symptoms at 6 and 12 months following the combined e-couch and ISG intervention [27]. Moreover, it provides support that there are additional benefits from participating in such an automated training program [28]. The lack of significant difference in findings between participants self-identifying as currently depressed versus those that did not is important. It suggests that these benefits may be conferred despite a lack of individual awareness and self-identification of depression. Finally, while it may be anticipated that participation in an ISG should offer stronger perceived benefits for loneliness and isolation, and improved ability to relate to others and cope with everyday stress this was not evidenced in the present study. However, further research examining established ISGs is needed to provide a more ecologically valid consumer evaluation of the benefits.

### Perceived Difficulties

Difficulties or problems were reported by some participants. The primary concern of those participating in the automated training program (e-couch) was that it was too impersonal. Although this represented a minority of participants (27.3%, 45/162) and the intervention aims to incorporate engaging graphical and written content, the findings suggests the need to focus on innovative strategies to increase the personalization of automated online interventions. Participants engaged with the ISG reported that they were upset and anxious that they could not help other members more. This may stem in part from rules of the board which required anonymity and are not a characteristic of all support groups. However, it may also be an intrinsic aspect of online support groups where participants lack face-to-face contact, or the asynchronous nature of the bulletin board format. Further, the concerns about not being able to assist more may arise regardless of the communication format (face-to-face or online). A peer supporter may not have the resources to solve all the problems of their peer with depression; this may be an inherent disadvantage of participation in ISGs for some potential participants.

### Limitations and Conclusions

Complementary to other more objective outcome evaluations [8-10,27], this study reports the experience and outcomes of this type of online intervention from the perspective of the participants. However, the findings must be considered in the context of several limitations. The analyses did not include participants who failed to provide follow-up data. They may have been less satisfied and less positive than those who were retained in the trial.

In addition, items used to assess the problems encountered and user confidence was adapted for the purpose of the study and as such has not been externally validated. While responses were accepted based on how the participant interpreted the meaning of the items, it is possible that individuals may differ in their understanding of terms such as prevention. Moreover, while tailoring the types of problems participants were asked about to the different interventions reduced the number of perceived irrelevant items being completed by participants, this approach offers limitations in that it makes comparisons between interventions difficult. Further research should consider assessing more generic issues that may be associated with all online interventions.

While further investigation of consumer reports across online mental health interventions is required, the results of the present study offer an important consumer perspective on program effectiveness which complements existing objective and empirical evaluations [27,28]. This enables a more complete understanding of the experiences of participants which may assist in the development and evaluation of future programs.

## Multimedia Appendix 1

Reported benefits of engaging in the online interventions.

## Multimedia Appendix 2

Reported help-seeking actions.

## Multimedia Appendix 3

Problems encountered by trial participants.

## Abbreviations

ISG: Internet support group
RCT: randomized controlled trial
