# Supplementary material for: Reducing Depression Through an Online Intervention: Benefits From a User Perspective
Source: JMIR Ment Health. 2016 Jan 8;3(1):e4. doi: 10.2196/mental.4356 (PMC4723724; doi:10.2196/mental.4356)
Supplement: Supplementary file 1 [file mental_v3i1e4_app1.pdf]

Reported benefits of engaging in the online interventions, by current depression status.

	Current depression		
	Yes	No	χ^2 *P*
	n/N (%)	n/N (%)	
**The website helped me to…**			
Discuss subjects that I felt unable to discuss before	66/147 (44.9)	26/70 (37.1)	.280
Feel encouraged and supported emotionally	73/148 (49.3)	31/67 (46.3)	.678
Feel less isolated and lonely	67/149 (45.0)	25/67 (37.3)	.293
Feel proud of myself for helping others	51/142 (35.9)	2265 (33.8)	.772
Learn more about depression and its treatment	111/148 (75.0)	57/72 (79.2)	.495
Seek professional help for my depression	28/141 (19.9)	6/60 (10.0)	.088
**How much do you feel the website helped you in:**			
Being productive at work	42/140 (30.0)	19/66 (28.8)	.859
Coping with everyday stress	81/147 (55.1)	34/68 (50.0)	.486
Enjoying life more	69/145 (47.6)	26/68 (38.2)	.201
Personal growth and understanding	88/146 (60.3)	36/67 (53.7)	.369
Reducing the emotional pain you were experiencing	61/143 (42.7)	30/68 (44.1)	.841
Reducing the symptoms of your depression	67/147 (45.6)	30/64 (46.9)	.862
Your ability to relate to others	76/146 (52.1)	28/69 (40.6)	.116
Your self-esteem and confidence	60/146 (41.1)	27/68 (39.7)	.847

Note. N values vary due to missing data. Percentage of respondents endorsing the statement as ‘agree-strongly agree’ or ‘made things a lot or somewhat better’ is indicated in parentheses.
