# Supplementary material for: Reducing Depression Through an Online Intervention: Benefits From a User Perspective
Source: JMIR Ment Health. 2016 Jan 8;3(1):e4. doi: 10.2196/mental.4356 (PMC4723724; doi:10.2196/mental.4356)
Supplement: Supplementary file 2 [file mental_v3i1e4_app2.pdf]

Reported help-seeking actions following participation, by current depression status.

	Current depression		
	Yes	No	
	n/N (%)	n/N (%)	χ^2 *P*
**Have you done something different because of the website?**			
Yes (total)	71/151 (47.0)	37/74 (50.0)	.899
Yes, given advice about depression to someone else	16/150 (10.7)	12/76 (15.8)	.269
Yes, sought help from a health professional	14/150 (9.3)	1/76 (1.3)	.022
Yes, sought more information	26/150 (17.3)	6/76 (7.9)	.055
Yes, tried a self-help treatment	43/150 (28.7)	23/76 (30.3)	.803

Note. N values vary due to missing data. Percentage of respondents endorsing the statement is indicated in parentheses
