# Supplementary material for: Reducing Depression Through an Online Intervention: Benefits From a User Perspective
Source: JMIR Ment Health. 2016 Jan 8;3(1):e4. doi: 10.2196/mental.4356 (PMC4723724; doi:10.2196/mental.4356)
Supplement: Supplementary file 3 [file mental_v3i1e4_app3.pdf]

Problems reported by trial participants, by current depression status.

	Current depression		
	**Yes**	**No**	
	n/N (%)	n/N (%)	χ^2 ***P***
**Were any of the following a problem for you as a result of using the e-couch program (or HealthWatch)?**			
Feeling bored	15/109 (13.8)	13/53 (24.5)	.089
Feeling frustrated	18/109 (16.5)	12/54 (22.2)	.376
Feeling more anxious	9/108 (8.3)	9/56 (16.1)	.133
Finding the program too impersonal	28/106 (26.4)	15/53 (28.3)	.801
**Were any of the following a problem for you as a result of using the WellBeing Board?**			
Feeling annoyed or upset by the comments made by other members on the board	5/62 (8.1)	2/31 (6.5)	.781
Feeling frustrated that I could not meet other members of the board in person	12/60 (20.0)	4/31 (12.9)	.399
Feeling upset that I couldn't help other board members more	17/61 (28.3)	10/31 (32.3)	.698
Feeling very anxious about other members on the board	12/61 (19.7)	2/31 (6.5)	.095

Note. N values vary due to missing data. Percentage of respondents endorsing the statement is indicated in parentheses
